# Supplementary material for: Cytomegalovirus-Associated Autoantibody against TAF9 Protein in Patients with Systemic Lupus Erythematosus
Source: J Clin Med. 2021 Aug 21;10(16):3722. doi: 10.3390/jcm10163722 (PMC8396997; doi:10.3390/jcm10163722)
Supplement: Supplementary file 1 [file jcm-10-03722-s001.zip › jcm-1336163-SI.pdf]

Cytomegalovirus-Associated Autoantibody against TAF9 Protein in Patients with Systemic Lupus Erythematosus

Yen-Fu Chen [1,†], Ao-Ho Hsieh [1,†], Lian-Chin Wang [1], Kuang-Hui Yu [1,*] and Chang-Fu Kuo [1,2,*]

1 Division of Rheumatology, Allergy and Immunology, Chang Gung Memorial Hospital, Taoyuan 333, Taiwan; patrichen0693@gmail.com (Chen, Y.-F.); mayitsu2006@gmail.com (Hsieh, A.-H.); lian.chin.wang@gmail.com (Wang, L.-C.)

2 Center for Artificial Intelligence in Medicine, Chang Gung Memorial Hospital, Taoyuan 333, Taiwan

* Correspondence: zandis@gmail.com (Kuo, C.-F); goutyu@gmail.com (Yu, K.-H); Tel.: +886-(03)-328-1200

† Equal contribution.

Supplemental tables

Table S1. The measurement of CMV IgG avidity in SLE patients with CMV IgG positive

CMV IgG positive (SLE, n=163)	Anti-CMV IgM	
	Positive (n=21)	Negative (n=142)
High avidity (>60%)	10	115
Intermediate avidity (30-60%)	11	27
Low avidity (<30%)	0	0

Table S2. The measurement of CMV IgG avidity in SS patients with CMV IgG positive

CMV IgG positive (SS, n=64)	Anti-CMV IgM	
	Positive (n=5)	Negative (n=59)
High avidity (>60%)	3	39
Intermediate avidity (30-60%)	2	20
Low avidity (<30%)	0	0

Table S3. The measurement of CMV IgG avidity in RA patients with CMV IgG positive

CMV IgG positive (RA, n=76)	Anti-CMV IgM	
	Positive (n=2)	Negative (n=74)
High avidity (>60%)	2	49
Intermediate avidity (30-60%)	0	25
Low avidity (<30%)	0	0

Table S4. The measurement of CMV IgG avidity in gout patients with CMV IgG positive

CMV IgG positive (Gout, n=70)	Anti-CMV IgM	
	Positive (n=3)	Negative (n=67)
High avidity (>60%)	3	53
Intermediate avidity (30-60%)	0	14
Low avidity (<30%)	0	0

Table S5. The measurement of CMV IgG avidity in AS patients with CMV IgG positive

CMV IgG positive (AS, n=52)	Anti-CMV IgM	
	Positive (n=2)	Negative (n=50)
High avidity (>60%)	1	30
Intermediate avidity (30-60%)	1	20
Low avidity (<30%)	0	0
